# A Literature Review on Perceptions and Practices Related to Healthcare and Nutrition Amongst the Residents of Urban Slums Across India

**DOI:** 10.7759/cureus.36654

**Published:** 2023-03-24

**Authors:** Akash Gajanan Prabhune, Usha Manjunath, Subodh S Satheesh

**Affiliations:** 1 Clinical Research, Institute of Health Management Research, Bangalore, IND; 2 Hospital and Health System Management, Institute of Health Management Research, Bangalore, IND; 3 Pharmacy, Institute of Health Management Research, Bangalore, IND

**Keywords:** healthcare inequality, knowledge attitude and practices related to health seeking, nutritional practices, health seeking behaviors, urban poverty

## Abstract

Urban regions are home to more than a billion people worldwide; by 2030, more than half of the world's population is projected to reside there. Many rural residents relocate to urban regions in pursuit of employment, better living conditions, and access to healthcare facilities. The study’s primary objective is to collate the findings related to perceptions, knowledge, attitude, and practices from studies across the urban slums in India related to healthcare and nutrition.

A systematic search of articles was conducted on the National Library of Medicine PubMed Portal Google Scholar, and J-Stor databases for published studies across the indexed journals. Academic social media sites like Academia.edu and Researchgate.org were also searched for grey literature. The inclusion criteria include studies conducted in Urban slums from 2010 to 2022, conducted amongst the Indian population within the Indian Geography, and focusing on documenting perceptions, knowledge, attitude, and practices. Exclusion criteria were cross-sectional surveys with quantitative questionnaires focusing on the prevalence of diseases and the burden of risk factors, literature reviews, systematic reviews, frameworks for implementation of specific interventions, and experimental study designs.

A total of 18 qualitative observational studies were included in the review and the findings related to knowledge, attitudes, and practices identified from the literature were summarized. The literature indicated adequate knowledge about nutrition and healthcare, and the barriers towards transitioning knowledge to practice were related to lack of resources, priorities around employment and income, and the attitudes towards change-making were usually based on convenience to access cost of service and availability of the services.

The review recommends further investment in research to understand the perceptions, patterns of nutrition, and health-seeking behaviours. Also, there is a pressing need to use the evidence for developing policies in line with the expectations of poor urban communities.

## Introduction and background

India is undergoing rapid urbanization and around a third of the total population live in cities, many rural residents relocate to urban regions in pursuit of employment, better living conditions, and access to healthcare facilities [1]. With the rapid urban expansion, a class of urban poor population has been rapidly rising around the cities. The Asian Development Bank defines Urban Poverty as “complex and multidimensional-extending beyond the deficiency of income or consumption, where its many dimensions relate to the vulnerability of the poor on account of their inadequate access to land and housing, physical infrastructure and services, economic and livelihood sources, health and education facilities, social security networks, and voice and empowerment” [2,3].

Urban poverty is multidimensional and its dimensions relate to the various forms of deprivations, disadvantages, and risks and are manifested in the lack of access of the poor in cities and towns to basic services, such as water and sanitation, shelter, and livelihood, and is becoming increasingly evident, in health, child mortality, education, social security, and empowerment and voice. Informal settlements or slums are the most visible form of urban poverty, these settlements often lack access to water and sanitation infrastructure, and the deficit infrastructure shows slum dwellers paying more for basic services such as clean water and electricity than residents living in adjacent fully serviced neighbourhoods [4]. Many urban poor communities rely on informal employment and are exposed to social poverty (lack of access to education, health, and social security).

A systematic review by Mireya Vilar-Compte et al. presented evidence on the effects of urban poverty on healthy eating, they summarize the challenges posed by urban poverty for adequate food access and nutrition as urban poor have an increased risk of consuming unhealthy and energy-dense foods associated with higher prevalence of overweight and obesity. Urban poverty was found to increase the chances of chronic undernutrition, leading to higher obesity prevalence in future stages of life, the review also suggested that psycho-social factors are important determinants of obesity through plausible biological links with stress and feelings of despair commonly experienced by people living in urban poverty [4]. The review concludes with “urban poverty leads to increased risk of poor nutrition outcomes including stunting, overweight and obesity".

The manifestation of urban poverty and food insecurity was highly correlated with poor health outcomes amongst children, women, and persons with disabilities. A study by Ashish Joshi et al. in urban slums of New Delhi found the odds of healthcare needs were fully or partially not met amongst the food insecure households (OR -1.19, P value 0.006) [5]. One of the key reasons is high out-of-pocket expenditure on healthcare amongst the surveyed families and prioritization of nutrition needs over healthcare needs [6].

Various studies conducted across the geographies have found a strong correlation between increased healthcare spending, food insecurity, and housing insecurity [6-10]. A geospatial study from the slums of Bangladesh showed 82% of healthcare providers within urban slums of Dhaka were private healthcare providers, 12% were public healthcare providers and 6% included not-for-profits and non-governmental organizations [11]. A qualitative study by Abdul Aziz et al. [12] indicated the inclination of the urban poor towards private healthcare providers, which mostly included informal healthcare which was due to inadequate healthcare facilities, unpleasant experiences at Government healthcare facilities, informal providers being the preferred option due to cultural linkages, dignified treatment, and flexibility offered in terms of house visits.

The information on behavioural patterns of the urban poor regarding dietary patterns, and health-seeking behaviours are spatially, temporally, and age-group-wise distributed across the literature [13-15]. Also, the evidence on health-seeking behaviour, dietary practices, and individual and family perceptions, are not clearly evident through a single source and thus, there is a need to collate and synthesize the information regarding perceptions and practices of poor urban communities across India and provide a focal understanding for research needs [16,17].

The study’s primary objective is to collate the findings from studies across the urban slums in India regarding perceptions and practices related to healthcare. The secondary objective is to identify common trends across various parts of the country and list the scope for further research.

## Review

Methods

A systematic search of articles was conducted on the National Library of Medicine PubMed Portal Google Scholar, and J-Stor databases for published studies across the indexed journals. Academic social media sites like Academia.edu and Researchgate.org were also searched for grey literature.

Keywords used for the search were urban poor, slum population, slums, preventive health, health perception, health-seeking behaviour, health-seeking patterns, nutritional practices, nutrition, and healthy eating. Appropriate Boolean operators “AND” and “OR” were placed.

The inclusion criteria used for selecting the studies

Studies conducted in Urban slums from 2010 to 2022, studies conducted amongst the Indian population within the Indian Geography, and studies focusing on documenting perceptions and related practices on health and nutrition.

The exclusion criteria included

**Figure 1:**
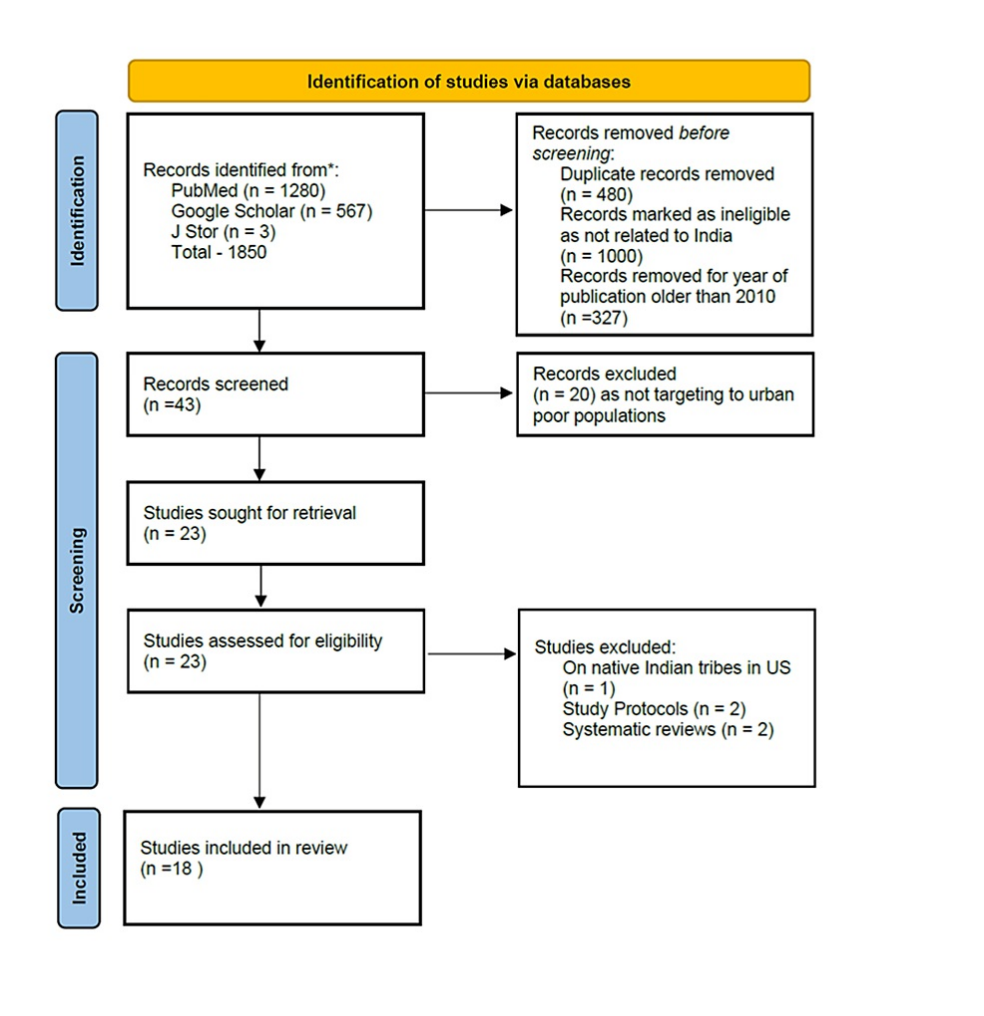
Prisma flow chart of the literature review process

Cross-sectional surveys with quantitative questionnaires focusing on the prevalence of diseases and burden of risk factors were excluded, Literature reviews, Systematic Reviews, and Frameworks for implementation of specific interventions were excluded and experimental study designs were excluded (Figure 1).

The search yielded 1850 articles, after screening for titles and abstracts 43 articles were selected. Most articles were excluded at this stage due to studies not being conducted in the urban slums and outside the Indian geography.

All 43 articles were screened for full-text versions by authors AP and KP, and 18 articles were included in the review. Two studies were excluded for not being conducted within Indian geography, two duplicates, and 26 studies were removed for not satisfying inclusion criteria (cross-sectional studies using only quantitative data for measuring the disease burden without any qualitative component, not including urban slums are target population), and one study was excluded as it was study protocol.

A detailed description of the reviewed 18 articles is presented in Table 1, the findings from the studies were clubbed under, knowledge of good health and nutrition, practices related to healthcare-seeking and eating habits, and attitudes related to change-making. Under each category, the findings were further stratified under the aegis of the socio-ecological model into the findings at the personal, interpersonal, family, community, and policy levels.

**Table 1:** Description of the studies included in the literature review BPL: below poverty line; AWWs: Anganwadi Workers; NMs: nurse mentors; ASHA: Accredited Social Health Activist

Author	Geography Covered	Study Objective	Study Design	Target Population	Thematic Focus
Mahua Patra and Satarupa Bandyopadhyay [1]	Urban Slums of Kolkata	What are the determinants of the choice of type of hospital (Public or Private)? What are the determinants of seriousness about health care seeking (more or less)?	Cross-sectional study using a semi-structured interview	BPL and Non-BPL households	Quality of care, and competing priorities and health needs
Misra et al. [13]	Urban Slums of Delhi	Determine the awareness and health-seeking practices related to common eye conditions.	Cross-sectional study using a semi-structured interview	Individuals aged 18 to 60 years and residing in notified slums	Sustaining life and health
Gaiha and Gadin [14]	Urban Slums of Delhi	To explore barriers and opportunities to participate in community-based health promotion initiatives among couples residing in slum settings. To investigate differences in sources and depth of health information between husbands and wives, as individuals as part of a couple.	Cross-sectional study using a semi-structured interview	Heterosexual couples aged between 20 to 60 years residing in notified slums	Sustaining life and health, and Competing Priorities and health needs
A. Gundewar and N. P. Chin [15]	Urban Slums of Mumbai	To identify how women from lower-income households leveraged their social capital to promote health within their households.	Mixed-method, qualitative study using in-depth, semi-structured, individual interviews	Female residents of notified slums aged over 18 years	Competing priorities and health needs
Das et al. [16]	Urban Slums of Kolkata	Understanding gender differences in respect of therapeutic choices in the slum context is crucial to developing appropriate policies to promote and provide suitable treatment sources for women’s and men’s requirements and thereby ensure better utilisation of health care facilities.	Cross-sectional study design using face-to-face in-depth interviews with a semi-structured questionnaire	Individuals between 16 and 46 and residents of slums	Competing priorities and health needs
Banerjee et al. [17]	Urban slums of Nagpur	To evaluate the implementation status of Urban Health and Nutrition Day (UHND) and to explore barriers and bottlenecks as perceived by community-level service providers.	Mixed-method study using in-depth interviews with a semi-structured questionnaire and checklist for quality assessment	NMs, ASHAs, AWWs, and an NGO representative working within the selected slums	Quality of care
Das et al. [18]	Urban Slums of Kolkata and Bengaluru	To get a more nuanced understanding through the narratives of urban slum dwellers of the diverse components of knowledge, neighbourhood, material culture and symbolic drivers of a place that interweave in shaping their patterned way of valuing health and managing in practice.	Cross-sectional study using a semi-structured interview	Individuals aged 18 and above and residents of selected slums	Sustaining life and health, and competing priorities and health needs
Das et al. [19]	Urban Slums of Kolkata and Bengaluru	To uncover the many facets of lay decision-making before future action is taken and the reasons underpinning illness-expressing behaviour among Indian urban slum dwellers.	Cross-sectional study using in‐depth semi‐structured interviews	Individuals aged 18 and above and residents of selected slums	Competing Priorities and health needs, and Quality of Care
Abdi et al. [20]	Urban Slums of Bengaluru	To identify, explore, understand, and prioritise the major priority health issues individuals residing in slums are facing.	Systematic review followed by semi-structured interviews with the stakeholders	Health professionals, community workers, technology experts, and experts with experience working on health projects in urban slums	Competing priorities and health needs
Černauskas et al. [21]	Urban Slums of Ahmedabad	To examine the factors affecting the choice of health care provider in a low-income setting	Discrete choice experiment establishing attributes and meaningful levels for each attribute; generating questionnaire through building of choice sets using experimental design; and survey application and analysis	Individuals above 18 years and residing in selected	Quality of care
P Sushama et al. [10]	Urban Slums of Bengaluru	To examine some practical challenges, faced working with two slum communities while trying to build participatory processes	Exploratory, Iterative design involving community visits, semi-structured interviews, prioritization workshops, community forums, photo voice activities, chulha-building sessions, and cooking trials.	Community volunteers working in selected slums	Quality of care
Vora and Shelke [22]	Urban Slums of Mumbai	To study the sociodemographic, dietary, and physical activity factors associated with obesity in the selected population, for the development of community-specific health education tool	Cross-sectional study with experimental design on selected participants	Women above 20 years of age	Sustaining life and health
Chopra et al. [23]	Urban Slums of Mumbai	To explore influences on the diet and physical activity of adolescents living in Mumbai slums, from the perspectives of adolescents and their caregivers	Cross-sectional design with focused group discussions	Adolescents (aged 10-12 and 15-17 years) and their caregivers	Competing priorities and health needs
L.A. Houghton et al. [24]	Urban Slums of Delhi	To characterize the feeding and caring practices of disadvantaged urban Indian children 12 to 24 months of age about the World Health Organization (WHO) recommendations	Cross-sectional study	Mothers of children aged 12 to 24 and residents of selected slums	Sustaining life and health
Athavale et al. [25]	Urban Slums of Mumbai	To assess the underlying barriers and facilitators for caregivers to implement recommended infant and toddler feeding practices in two urban communities	Cross-sectional study with in-depth semi-structured interviews	Mothers of children aged 6 to 24 and residents of selected slums	Sustaining life and health
Ravindranath et al. [26]	Urban Slums of Ahmedabad	To categorize the current nutritional status of children under the age of five and determine the underlying causes of poor nutritional outcomes	Cross-sectional study	Parents of children under age 5 and residents of the selected slums	Competing priorities and health needs
Kumar et al. [27]	Middle-class residents of Mumbai and Kochi, including from slums	To understand how urban Indian middle-class consumers define and perceive processed food. To understand the changes in food choice as well as explore the reasons behind it.	Cross-sectional design with focused group discussions and semi-structured interviews	Individuals aged 40 to 65 and having an annual income above 200,000	Sustaining life and health
Kusuma et al. [28]	Urban Slums of Delhi	To understand the awareness, access, and utilization of health insurance and determinants of possession of health insurance among the urban poor in Delhi	Cross-sectional study using pretested, interviewer-administered questionnaire	Adults residents of selected slums	Competing priorities and health needs

Results

The detailed characteristics of the studies included in presented in Table 1. We classified the studies across the thematic areas. Three thematic areas were defined based on the broader themes that arise during the literature review.

Theme 1 was about sustaining life and health; focusing on personal and interpersonal levels of the socio-ecological model and concerning personal and family choices, answering why, how, and who makes the choices regarding health seeking. Theme 2 was about competing priorities and health needs; focused on the community level of the socio-ecological model and concerning how cultural norms shape health-seeking behaviour and nutritional behaviour. The third theme was on quality care; focused on the societal level of the socio-ecological model and concerning access, availability, and affordability of healthcare services.

Competing priorities and health needs were the most common themes 52% (n=9) indicating the literature was focused on health-seeking behaviour and nutritional practices of the target population. Sustaining life and health was the second common theme 41% (n=7) with a focus on choices related to health and nutrition. The theme of quality of care was least common with 29% (n=5) articles focusing on access, availability, and affordability of healthcare services.

Geography-wise the studies were restricted to metro cities and major cities with Mumbai having covered by five studies, followed by Delhi, Kolkata, and Bengaluru each reporting four studies. Ahmedabad, Nagpur, and Kochi were other major towns wherein at least one study was conducted.

Knowledge of good health and nutrition

Knowledge of good health and nutrition referred to the knowledge of the respondents related to good practices like handwashing, use of toilets, clean drinking water, eating fresh fruits and green leafy vegetables, exercise, and physical activity and their impact on overall health status.

The study by Gaiha et al. focused on adolescents and their caregivers (parents) unfolded interesting insights into the knowledge related to a healthy diet [14]. All caregivers were having knowledge of high salt, high sugar, and high-fat food items were unhealthy. All the adolescents were also having the same knowledge that food containing high salt, and sugar fats increase weight, and causes acne. Another relevant finding was the caregivers were not having a clear understanding of a balanced diet or a nutritious diet. The general understanding amongst both adolescents and caregivers was food cooked at home is usually healthier than the one that is cooked outside.

A study conducted by Athavale et al. across the Mumbai slums amongst young mothers indicated clear knowledge of exclusive breastfeeding from healthcare providers, there was however conflicting information on family elders regarding exclusive breastfeeding [15]. The study also recorded the knowledge about hygiene and safe cooking practices which were found to be adequate as per national guidelines on young infant and child feeding practices.

Another study by Gaiha and Gadin explored the knowledge amongst young married couples on topics related to prevention, management, care, support, treatment, and rehabilitation: the questions were focused on food habits and nutrition; water, sanitation, and hygiene; physical activity; tobacco consumption, reproductive health (family planning methods); drug/alcohol abuse; Human Immunodeficiency Virus/Acquired Immunodeficiency Syndrome (HIV/AIDS); mental illness; newborn health and heart disease [16]. The source of knowledge for men was mass media, including radio, television, information, education, and communication (IEC) materials. For women, the source of knowledge was interpersonal communication (IPC) by elders, parents, relatives, school/children’s school, peers, traditional healers, and employers. The most trusted source of information for the couples was the elder women in the house.

A study by Abdi et al. explored the knowledge of participants from slums to identify and rate common diseases based on health priorities [17]. Diabetes followed by hypertension was the most common non-communicable disease recognised and stated to benefit from early screening. Dengue and diarrhoea were common infectious diseases with knowledge of risk factors, how to seek treatment, and disease course. Under the maternal and child diseases the knowledge on anaemia, and malnutrition was seen to be most comprehensive.

The study by Misra et al. amongst elderly participants living in urban slums indicated 50% of people were having knowledge about cataracts, related symptoms, and treatment options, and the knowledge was positively associated with years of schooling, and employment [18]. Males were more likely to present with knowledge about cataracts compared to women.

Overall, the studies reviewed were focused on a particular disease, age group, and knowledge of nutrition. The study by Kumar et al. comparing the food choices among the non-slum and slum residents indicated similar levels of knowledge across the populations about the consumption of processed and packaged foods [19]. The studies had limited exploration done to link health habits like exercise, hand washing, and boiling water before drinking, with health conditions.

Practices related to the healthcare-seeking and eating habits

The studies by Gaiha and Gadin, Gundewar and N. P. Chin, and Das, indicated that most respondents defined ill health as related to physical aspects (pain, swelling, uneasiness) and physical aspect was the most common reason for the visit to the health care provider. The study by Das reported that mental aspects of health were known to the participants, but was dismissed as something that can be managed with rest without the need to seek care from professionals. The Gaiha and Gadin study also cited the reason for dismissing mental health as being a lack of awareness about mental health conditions, the paucity of time to focus on something outside the physical health paradigm, and mental illness is highly stigmatized.

The series of studies by Das et al. across the urban slums of Kolkata, and Bengaluru indicated statistically significant differences related to health-seeking behaviours across the male and female respondents. The preferred choice of healthcare providers for males was pharmacists who would give tablets listening to their chief complaints and the transaction would not need any clinical examination or laboratory tests. Interestingly if the treatment sought from the pharmacist was not effective males preferred visiting private clinics and choose not to follow up with the pharmacist. The underlying reason for the behaviour cited is a preference for quick treatment, most studies reported males considered falling ill as a burden in terms of wage loss due to absenteeism, and thus preferred quick remedies. This was also reflected in the choice of therapy wherein men preferred modern medicine as its quick for gaining symptom relief. For serious health issues, the men preferred Government Hospitals.

The women had different practices with preferences for local traditional healers and herbalists for minor ailments. However, when the treatment would not yield desired results, the women would prefer the local traditional healer or herbalist to recommend the healthcare provider for further referral and would also follow up with the local traditional healer or herbalist regarding the treatment provided by a referred healthcare provider [21]. This was mainly because of the trust established between the local healthcare provider and the women seeking treatment. The women preferred healthcare providers who knew their language had similar cultural understanding, explained the cause of disease and the treatment plan in detail, and more importantly, were well-versed with the cultural practices of the community.

Both men and women, preferred government healthcare services for maternal and child health including immunization as they were able to seek the benefits of various government schemes.

For the elderly group, the study by Misra identified a preference for charitable trusts or NGOs for the treatment of cataracts as the trusts or NGOs organising camps would arrange for transportation and even provide free surgery for the residents [22]. This was also strengthened by the good reviews shared by their friends and family members who had earlier sought treatment or surgery for cataracts from screening camps.

The study by Černauskas explored the likelihood of factors that determine the choice of healthcare providers, the factors examined were provider cost and type, distance to facility, the attitude of the doctor and staff, familiarity with the doctor, appropriateness of care, the results indicated for elderly patients nearness to healthcare facility was the key determinant for provide choice, for women and elderly women familiarity with the doctor and friendly attitude towards the patient. Young and formally educated participants had a strong preference for a friendly attitude of doctors and staff and appropriateness of care.

The study by Chopra et al., on adolescents, indicated caregivers and adolescents both expressed the need to undertake physical activity and a nutritious diet for good health, however, in practice they were not able to dedicate time to physical activity and continued eating unhealthy items like high salt, high sugar, and high fat containing items [23].

Attitude related to change-making

The attitude related to change-making explored the factors or the needs mentioned by the respondents to enable them to make positive changes related to diet and health. Most studies reviewed have not explored the attitude toward change-making directly. However some of the responses documented in the publication hint towards factors like: for male residents of the slums, across Delhi [14] and Mumbai [15], the quick service provisions were the key factor in shifting across various healthcare providers, they prefer shorter waiting times and medicines that can assure quicker recovery, thus the confidence of a healthcare provider also accounts to change making determinants. In the case of older residents closeness to the healthcare provider was a key factor in change-making, they preferred to change to the closest healthcare provider while relaxing on cost and waiting time. For pregnant women and young mothers, clear instructions in written and explained by a nurse and/or Accredited Social Health Activist (ASHA) regarding young infant feeding practices was a clear change maker in terms of adopting WHO child feeding practices [24]. In the case of breastfeeding mothers, the reassurances and encouragement from doctors or nurses regarding breastfeeding practices helped them to continue the change [25]. Regarding food choices, many participants mentioned that TV advertisement has played a crucial role in changing food habits, the TV advertisement tempts them to eat outside [26].

Mind map

**Figure 2:**
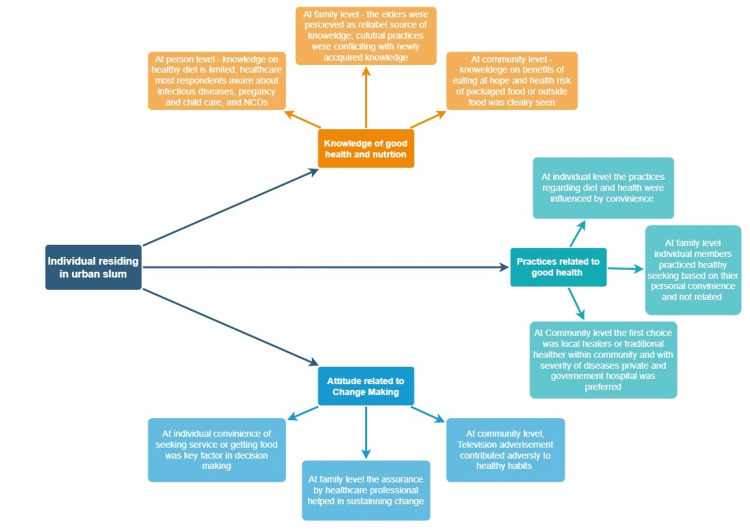
Mind map on the study results contextualized on social-ecological model results

The mind map was constructed to summarise the findings of the review and contextualized in terms of the socio-ecological model, at the individual level the knowledge was about healthy diet, and nutritious food and its linkages to health were similar to contemporary populations across the non-slum residents, while practising the convenience of getting a food or healthcare service deviated from practising as per knowledge, and the same was seen across the change-making if the convenient availability of health food or healthcare service is provisioned individuals were willing to change (Figure 2).

Across the families, cultural practices did cloud the knowledge of good health and diet and also influenced practices, but most family members perceived convenience as a key determinant of practice. To sustain the change regarding diet and health positive reinforcement from providers like doctors, and nurses were a key criterion.

At the community level, the knowledge remained good, the practices were dictated by personal preferences, and television was considered a key barrier that prevented change to healthy habits.

Discussion

The current review is focused on perceptions of people living in urban slums and how those perceptions have translated into practices that impact the health and nutrition of the population at large. During our review of various articles, a limited number of articles were exploring the linkages between diet and health.

A systematic review by Vilar-Compte et al. presents the global perspective of urban poverty and has developed a conceptual framework with “Food Security” as a central concept branching out to issues like employment, income at the personal level, income, housing conditions at the family level, food pricing, neighbourhood characteristics at the community level [27]. The populations that lack access to healthy meals and who consume the ones of poor quality are at greater risk. Similar connections were identified from the current review for health and nutrition.

Another review by Harpham et al. presented a multidimensional overview of urban poverty and its association with nutrition and health [28]. The paper noted the need to move from ill health to preventive health, considering the current research has a limited focus on positive action. Slum residents are deprived of good health for a number of reasons, including limited access to healthcare services and inadequate knowledge of healthcare issues. The key areas of research should be targeted to answer the questions like why are some individuals, households or groups better able to cope with these conditions, and consequently have better health. We need to know what to strengthen among low-income urban populations to protect and promote their health, and how to strengthen it. The current study also aligns with the conclusion of the Harpham paper stating the need to look for positive actions to be further studied and explored.

The review by Kusuma and Babu stated the urban poor communities have similar knowledge regarding diseases and nutritional facts, they are also well aware of the risk factors for infectious diseases and non-communicable diseases, the key barrier to conversion of knowledge into practices is a lack of resources (monetary and availability of services) [29]. The current study also observed the barriers to translating knowledge into practices are usually due to focus on work, lack of money, and service delivered at an inconvenient time. The use of technological breakthroughs in the promotion of health education can help to reach a larger demographic.

The strengths of the current study are that the study analyses the information from the perspective of individuals, families, and communities and tries to establish linkages between the perceptions and practices related to healthcare and nutrition of any individual living in urban poor areas. This presents more substantial data on how knowledge, attitudes, and practices are shaped across the communities and provides scope for additional research. The study has its own limitations one being the information is published from metro cities and lesser information from non-metro or tier II and III cities is available.

From this review, it was observed that there is a need to undertake further research on understanding how knowledge about nutrition and health can be converted into actionable steps. A clear framework needs to be developed on the perceptions of the urban poor populations from India on good health and well-being, the probable enablers and barriers, and mechanisms to address the barriers. For the policies to better reflect the requirements of urban poor communities, there is a need to establish suitable evidence.

## Conclusions

The knowledge among the residents of the urban poor communities was clearly represented across the literature. There was a clear indication that despite having clear knowledge of nutrition and health-seeking the practices were influenced by convenience, and availability of monetary resources. The factors affecting the practices have been similar across India. For example, the adult males preferring quicker turnaround time across the OPDs have been recorded from Mumbai and Delhi.

There is limited literature on perceptions and patterns about the services delivered through public health systems. Although it is clearly evident that across the slums public healthcare providers like Accredited Social Health Activists (ASHAs), and Anganwadi Workers are important stakeholders. The role of community agencies in monitoring government service delivery is not explored in depth through literature.

There is a pressing need to develop a separate category for residents of urban slums in terms of policy, research initiatives and developing interventions to promote preventive health behaviour and improve dietary practices through.
